# Exploring Somali-born women’s experiences with contraceptive services in Sweden through a reproductive justice lens

**DOI:** 10.1080/26410397.2026.2667110

**Published:** 2026-05-08

**Authors:** Anna Wängborg, Marie Klingberg-Allvin, Sahra Saidarab, Cristina A. Mattison, Elin C. Larsson, Helena Kilander

**Affiliations:** a PhD student, Department of Women’s and Children’s Health, Karolinska Institutet, Stockholm, Sweden.; b Professor, Department of Women’s and Children’s Health, Karolinska Institutet, Stockholm, Sweden; c Research Assistant, Department of Women’s and Children’s Health, Karolinska Institutet, Stockholm, Sweden; d Research Affiliate, Department of Women’s and Children’s Health, Karolinska Institutet, Stockholm, Sweden; e Principal Researcher, Department of Women’s and Children’s Health, Karolinska Institutet, Stockholm, Sweden; Principal Researcher, Department of Global Public Health, Karolinska Institutet, Stockholm, Sweden; Program Officer, Centre for Epidemiology and Community Medicine, Region Stockholm, Stockholm, Sweden; f Researcher, Department of Women’s and Children’s Health, Karolinska Institutet, Stockholm, Sweden; Associate Professor, WHO Collaborating Centre, Karolinska University Hospital, Stockholm, Sweden; Associate Professor, Jönköping Academy for Improvement of Health and Welfare, School of Health and Welfare, Jönköping University, Sweden

**Keywords:** child spacing, contraceptive services, immigrant, improvements, midwifery, person-centred contraceptive counselling, reproductive justice

## Abstract

Timely and equitable access to contraceptive services is a key component of sexual and reproductive health and rights. Evidence shows that immigrant women in high-income countries often receive inadequate contraceptive counselling. There is also limited knowledge about how contraceptive services can best be organised within health systems to meet their needs. In Sweden, the Somali population is one of the country’s largest immigrant groups, facing a higher risk of adverse pregnancy and childbirth outcomes compared to Swedish-born women. This study aimed to gain a deeper understanding of Somali-born women’s perceptions of and experiences with contraceptive services in Sweden. Eight focus group discussions were conducted, with a Somali-speaking moderator, and data were analysed using Braun and Clarke’s reflexive thematic approach. All participants (*N* = 60) were Somali immigrants who had given birth in Sweden. Two main themes were constructed: (1) Factors shaping reproductive choices: community, household, and partner influences, and (2) Women’s perspectives on contraceptive services. The findings suggest opportunities to advance reproductive justice in contraceptive services in Sweden. Incorporating women’s preferences and needs is essential to establishing person-centred contraceptive services, aligning with Sweden’s health system priorities. Services must adapt to and reflect clients’ experiences to avoid being shaped by preconceived notions and intersecting power dynamics. Our findings have implications for moving towards reproductive justice in the delivery of sexual and reproductive health services.

## Plain language summary

Access to good-quality contraceptive services is an important part of sexual and reproductive health and rights. Research shows that immigrant women often do not get enough support or information about contraception. In Sweden, Somali-born women face more health risks during pregnancy and childbirth compared to Swedish-born women. This study looked at how Somali-born women in Sweden experience and think about contraceptive services. We conducted eight group discussions with 60 women, led by a Somali-speaking moderator. From the conversations, we found two main themes: (1) factors shaping reproductive choices and (2) women’s perspectives on contraceptive services. The findings show that the health system needs to listen better to women’s needs and experiences to improve contraceptive services. To be fair and respectful, services should avoid stereotypes and take each woman’s situation into account. These findings can help improve sexual and reproductive healthcare for others in similar situations.

## Résumé

Un accès ponctuel et équitable aux services de contraception est un élément essentiel de la santé et des droits sexuels et reproductifs. Il est démontré que les immigrées dans les pays à revenu élevé reçoivent souvent des conseils inadaptés en matière de contraception. De plus, les connaissances sur la manière d’organiser au mieux ces services au sein des systèmes de santé pour répondre à leurs besoins sont limitées. En Suède, la population somalienne représente l’un des plus importants groupes d’immigrés du pays, mais elle est confrontée à un risque plus élevé de complications pendant la grossesse et l’accouchement que les femmes nées en Suède. Cette étude visait à mieux comprendre la perception et l’expérience de femmes nées en Somalie en matière de services de contraception en Suède. Huit discussions par groupe d’intérêt ont été menées avec un modérateur de langue somalienne, puis les données ont été analysées au moyen de l’approche thématique réflexive de Braun et Clarke. Toutes les participantes (*n* = 60) étaient des immigrées somaliennes ayant accouché en Suède. Deux thèmes principaux sont apparus: (1) les facteurs conditionnant les choix en matière de procréation: influences de la communauté, du ménage et du partenaire; et (2) le point de vue des femmes sur les services de contraception. Les résultats suggèrent des pistes d’amélioration de la justice reproductive dans les services de contraception en Suède. L’intégration des préférences et des besoins des femmes est capitale pour mettre en place des services de contraception centrés sur la personne, conformément aux priorités du système de santé suédois. Ces services doivent s’adapter à l’expérience de leurs clientes et en tenir compte pour ne pas être influencés par des idées préconçues et des rapports de pouvoir croisés. Nos conclusions contribuent à faire avancer la justice reproductive dans la prestation des services de santé sexuelle et reproductive.

## Resumen

El acceso oportuno y equitativo a los servicios de anticoncepción es un componente clave de la salud y los derechos sexuales y reproductivos. La evidencia muestra que las mujeres inmigrantes en países de altos ingresos a menudo reciben consejería anticonceptiva inadecuada. Además, los sistemas de salud tienen conocimiento limitado sobre la mejor manera de organizar sus servicios de anticoncepción para atender las necesidades de esas mujeres. En Suecia, la población somalí es uno de los grupos de inmigrantes más grandes del país, pero enfrenta un mayor riesgo de resultados adversos del embarazo y el parto comparado con las mujeres nacidas en Suecia. Este estudio pretendía entender mejor las percepciones y experiencias de las mujeres nacidas en Somalia con relación a los servicios de anticoncepción en Suecia. Se realizaron ocho discusiones en grupos focales, con un moderador de habla somalí y se analizaron los datos utilizando el enfoque temático reflexivo de Braun y Clarke. Todas las participantes (*N* = 60) eran inmigrantes somalíes que habían dado a luz en Suecia. Se elaboraron dos temas principales: (1) factores que influyen en las decisiones reproductivas: influencias de la comunidad, la vivienda y la pareja; y (2) perspectivas de las mujeres sobre los servicios de anticoncepción. Los hallazgos indican oportunidades para promover la justicia reproductiva en los servicios de anticoncepción en Suecia. Incorporar las preferencias y necesidades de las mujeres es esencial para establecer servicios de anticoncepción centrados en la persona y alineados con las prioridades del sistema de salud sueco. Los servicios deben adaptarse a, y reflejar, las experiencias de las usuarias para evitar ser moldeados por ideas preconcebidas y dinámicas de poder interrelacionadas. Nuestros hallazgos tienen implicaciones para avanzar hacia la justicia reproductiva en la prestación de servicios de salud sexual y reproductiva.

## Background

Sexual and reproductive health (SRH) services are essential to good health and are a basic human right.^1^ Timely and equitable access to contraceptive services is a crucial aspect of sexual and reproductive health and rights (SRHR), as it benefits not only the health and well-being of individuals but also the broader community's health and development.^1^ Increased migration has greatly impacted individuals, health systems and societies,^2,3^ and immigrants face inequities in SRHR during^4^ and after migration due to both social and structural factors.^5–8^

Contraceptive services are defined as providing counselling, prescribing and administering contraceptive methods, such as intrauterine devices (IUD) and subdermal implants.^9,10^ Studies from high-income countries have shown that women with migratory experience, including refugee and asylum-seeking women, face barriers to contraceptive and other sexual and reproductive health services, including limited knowledge, language and communication challenges, and difficulties accessing services.^11–14^ Research on contraceptive counselling further suggests that services do not always respond adequately to women’s preferences, autonomy, and need for respectful, non-judgemental care.^15–17^

Although the Swedish government promotes equal SRHR among the entire population,^18^ immigrant women continue to have less access to contraceptive services than Swedish-born women.^11,19^ In Sweden, midwives provide most contraceptive services,^20^ which are available to individuals of all ages and free at the point of care for Swedish citizens and residents with a residence permit, including asylum seekers. Swedish midwives hold an ethical and professional responsibility to uphold the dignity, integrity, and autonomy of each individual. Their practice is guided by an ethical and evidence-based approach that respects human rights, autonomy and justice and responds to individuals’ social and cultural contexts.^21^ While midwives play a central role in the direct provision of contraceptive services, it is important to distinguish these encounters from the broader health system within which such services are organised. In this paper, we distinguish between healthcare services, the direct provision of counselling and clinical encounters, and the broader health system. The health system is an interacting set of governance, financial and delivery arrangements and implementation strategies that determine who provides care, where it is provided, how it is organised, and how it is financed.^22,23^ From a reproductive justice perspective, this distinction underscores that reproductive autonomy depends not only on individual encounters but also on systemic conditions, including accountability, that shape access, quality, and equity in services.^24^

A previous intervention study in Sweden found that postpartum use of effective contraceptives among immigrants increased following a project designed to improve contraceptive services for this population.^9^ However, the study did not report how the women perceived the counselling or whether they could make autonomous reproductive decisions.^13^ In this study, autonomy refers to the ability to make independent, informed choices, free from external pressure, that reflect one's personal reproductive goals and values.

Somali-born women in Sweden, one of the larger immigrant groups,^25^ experience worse mental and physical health-related quality of life than Swedish-born women,^26^. Furthermore, they have a higher risk of negative health outcomes for mothers and infants during pregnancy and childbirth.^27^ There is a limited understanding of the structural and systemic barriers and facilitators affecting immigrant women’s access to contraceptive services in Sweden. In response, there is a growing call for person-centred, culturally safe, and equity-driven contraceptive services. Understanding the experiences and perspectives of Somali-born women is essential to inform such improvements and to strengthen accountability within contraceptive services. This study seeks to address this gap by exploring the experiences of using contraceptive services among Somali-born women, with the aim of ensuring that their diverse needs and preferences are represented in the research evidence.

## Aim

This study aimed to deepen the understanding of Somali-born women’s perceptions of and experiences with contraceptive services in Sweden. The findings aim to inform improvements in service delivery, guide policy and practice, and contribute to advancing equitable healthcare in line with national priorities.

## Theoretical framework

This study is grounded in the reproductive justice framework, which uses an intersectional lens to examine how overlapping systems of oppression, such as ethnicity, gender, and socioeconomic status, shape experiences of sexuality and reproduction.^24,28,29^ The reproductive justice framework highlights the interconnected power dynamics that influence access to rights, particularly for persons in marginalised situations. Reproductive justice emphasises the importance of considering multiple factors and ensuring accountability when addressing inequities. This broader approach avoids a narrow focus on single issues, such as a lack of client knowledge or cultural insensitivity among healthcare providers, which can lead to ineffective solutions when organising healthcare services. Instead, reproductive justice directs attention to organisational and systemic barriers within health systems, as well as the power imbalances that affect access to quality care, including informed decision-making and reproductive autonomy.^24,28^

The reproductive justice movement started in the 1990s in community-based organisations led by women of colour in the United States (US) as the dominant reproductive rights movement at the time was led by middle-class white women and excluded the specific needs of Black, Indigenous and people of colour.^24^ Reproductive justice is achieved when individuals can exercise core rights: to bodily autonomy, to have children, not to have children, and to parent in safe, supportive environments free from coercion and violence.^24^ Its adaptable framework is widely applied across contexts and enables critical, reflexive examination of power dynamics in research.^30,31^ Ross^27^ also argues against restricting reproductive justice to US or Black communities, describing it as a “faulty presumption … that Black women cannot create universal praxis and theory applicable beyond our social location” (p. 301).^29^ In the Swedish context, it allows for examination of how systemic barriers such as migration status, access to healthcare, and cultural expectations influence Somali-born women’s contraceptive autonomy choices and experiences. Intersectionality theory served as the lens guiding how multiple intersecting social identities, such as ethnicity, gender, and socioeconomic status, shape participants’ experiences with contraceptive services.^32,33^ The reproductive justice framework is a structural, intersectional approach^24,28,34^ used in this discussion to contextualise and interpret these findings in relation to broader structural and systemic factors. This study's ontological and epistemological framework was rooted in constructivism, which views knowledge as actively constructed through individuals’ interactions with their environment. Constructivism is well-suited for understanding experiences.^35^

Using an inductive approach, the reproductive justice framework was applied in the discussion section of this study. By using reproductive justice as both an ethical foundation and an interpretive lens, we challenged the traditional focus on individuals as isolated units of study and instead considered the broader organisational and systemic factors at play. Reproductive justice positions the voices of communities in marginalised situations as central sources of knowledge, and centres narrative sharing as a way to document and legitimise lived realities that are often excluded from mainstream research.^24,28^ Specifically, reproductive justice helps us explore structural barriers within the Swedish health system and their impact on women’s access to contraceptive services. Our intersectional analysis may capture differences within the group, yet we also present women’s accounts collectively to expose structural and systemic barriers in contraceptive services that must be addressed to improve equity.^24,28^ This framework enables us to make visible the experiences of Somali-born women as they navigate these barriers, ultimately informing more equitable, person-centred and culturally responsive services.

## Design and methods

This inductive, qualitative study employed focus group discussions (FGDs) for data collection, to facilitate the sharing of personal experiences and support discussions of sensitive topics among peers with similar backgrounds.^36,37^ During the process, the realities expressed in the dataset were interpreted through a critical approach, utilising both semantic and latent coding.^38^ The data reporting follows the Consolidated Criteria for Reporting Qualitative Research (COREQ) checklist (see Supplementary Material).^39^

### Study setting

The study was part of a broader intervention study, the IMPROVE-it-project,^10^ which employs a quality improvement collaborative approach. The project incorporated co-design and customised interventions aimed at enhancing post-partum contraceptive services for immigrants in Sweden. The findings of this qualitative study were incorporated into workshops co-designed with midwives and immigrant women to develop contraceptive services meeting the target groups’ needs and preferences.

Sites for our study were selected in collaboration with doulas and health informants, who also assisted the research team with the recruitment of participants through face-to-face community invitations.

### Participants and data collection

Study participants were recruited via purposive sampling^40^ from two of the three regions of Sweden that were part of the IMPROVE-it project.^10^ Participants were eligible if they were (1) ≥ 18 years old, (2) born in Somalia, and (3) had given birth in Sweden.

Prior to data collection, two researchers conducted site visits to community settings where Somali-born women gather, including ongoing projects in both regions, to establish trust, familiarise themselves with the context, and facilitate participant recruitment. These informal meetings, held both in person and digitally, introduced the study, discussed its relevance, and provided information on participation, ethical approval, and consent (available in Swedish and Somali), as well as giving the women the opportunity to ask questions. Digital FGDs allowed women from different regions to participate and were preferred during Ramadan. Participants could choose the type of participation, ensuring flexibility and respect for preferences.

Each FGD began with study information, followed by verbal and written consent. Childcare was offered during FGDs, provided by staff familiar to the women. The research team (AW, HK, MKA, ECL and SS) developed a topic guide (Supplementary File 1). It covered themes including pregnancy planning and contraception, knowledge and use of methods, partner involvement, access to services, and women’s preferences for how, when, where and by whom counselling should be provided. The guide was pilot-tested in one FGD with Somali-speaking women, moderated with the help of an interpreter; this data were excluded. Based on this pilot, the guide was revised, and it was decided that all FGDs should be moderated by a Somali-speaking midwife, rather than using interpreters.

Eight FGDs (Table 1) were conducted between February and October 2022, with 60 women in total (Table 2). Seven FGDs took place in one region (six face-to-face, one via Teams) and one via Teams in another region. Groups included 4–12 women (median: 7) and lasted 22–76 minutes (median: 49). All FGDs were moderated by a Somali-speaking midwife and co-author (SS), assisted by AW, who took notes. Both face-to-face and digital FGDs were conducted, drawing on research on the usefulness of digital platforms.^41^

**Table 1:** Characteristics of the focus group discussions included in this study

FGD no/Setting	Date	Number of participants	Age (Range)	Years in Sweden (Range)	Years of education (Range)	Duration of FGD (Minutes)	Region
1 Community based	Feb 22	10	26–45	4–16	2–15	68	Västra Götaland
2 Online	April 22	12	28–44	4–14	1–12	76	Västra Götaland
3 Community-based	Sept 22	4	32–33	6–10	3–9	55	Västra Götaland
4 Community-based	Sept 22	5	29–35	5–18	1–10	44	Västra Götaland
5 Community-based	Sept 22	4	32–48	7–22	12–18	28	Västra Götaland
6 Community-based	Sept 22	7	24–37	4–14	4–13	22	Västra Götaland
7 Community-based	Sept 22	11	28–42	4–15	1–12	38	Västra Götaland
8 Online	Oct 22	7	24–42	9–31	5–15	65	Jönköping

**Table 2:** Characteristics of participants in the FGDs (*N* = 60)

Characteristics	Value
Age: mean	34
Years of education: mean	8
Years in Sweden: mean	10
Current occupation	
*Working*	*n* = 13*
*Student*	*n* = 12
*Parental leave*	*n* = 32
*Looking for work*	*n* = 3
*Did not answer*	*n* = 1
Marital status	
*Single*	*n* = 5
*Married*	*n* = 55
Number of children: mean	4
Experience of contraceptive use	*n* = 41
Experience of induced abortion	*n* = 14
*One participant reported both working and studying	

### Data management

The data were audio-recorded and stored on a two-factor authentication-encrypted drive accessible only to the research team. A research assistant transcribed the data verbatim and then anonymised and translated it from Somali to English. SS double-checked and controlled the quality of the translation. Signed consent forms were stored in a safe, locked place, and recordings of verbal consent were stored on a two-factor authentication-encrypted drive.

### Data analysis

The study was inductive, and the data were analysed using Braun and Clarke’s reflexive thematic analysis with six phases guiding the iterative process, including familiarisation, coding, generating initial themes, developing and reviewing themes, refining, defining, and naming themes, and writing up.^38,42^ The data were analysed using NVivo software. During familiarisation, author AW read the transcripts several times with the study’s aim and research questions in mind and created mind maps for each FGD. Next, AW, SS and ECL invited participants (N=12) from the FGDs to take part in member reflections on the preliminary interpretations; this resulted in no changes in the analysis. This was carried out twice during the analysis, and reimbursement was included. This process was designed to foster community trust, aligning with the reproductive justice framework,^24,28,34^ and to extend participants’ autonomy in the research process.^43^

**Figure 1:**
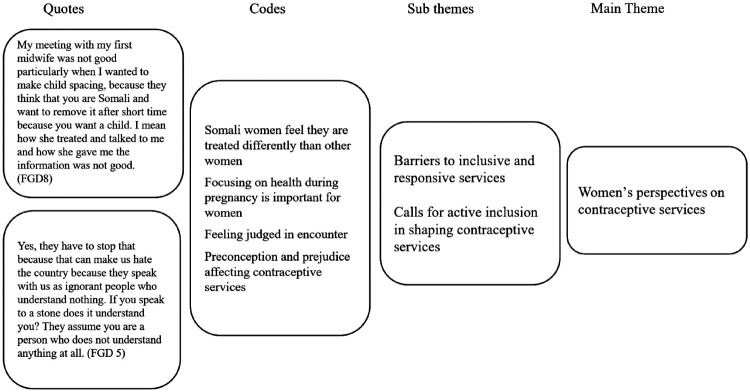
Example of audit trail

The initial coding was conducted by AW, and it was iteratively discussed with the rest of the team (HK, MKA, SS, ECL, and CM). Guided by SS, who speaks Somali, the research team discussed and reflected on the role of interpretation and the meanings of words and phrases in Somali. After the data were systematically reviewed and fully coded, the codes were collated to include depth, patterns and diversity. The next phase involved creating initial themes and subthemes, which entailed shifting from interpreting individual parts of the dataset to identifying overarching meanings within the coded transcripts based on FGDs. The initial themes and subthemes were identified through coding and relevant quotations, enabling the research team to accurately name them and create a thematic map, as exemplified in the audit trail (Figure 1). Next, the team described the themes and how they together told a narrative that represented the data, and verified that each theme contributed coherent data. The results were again reflected on with participants from the FGDs. At the end of the analysis, the results were written up and thoroughly checked in accordance with the study’s aim, and the themes and subthemes were revised accordingly.

### Researcher characteristics and reflexivity

Throughout the research process, the team discussed and reflected on their preconceived notions and biases, as well as the role of researchers in targeting persons perceived to be in marginalised situations. In addition, as the research team is primarily White, it was imperative to employ a framework designed by women of colour, such as reproductive justice, as an ethical approach and a lens in our discussion section. The research team comprised one Spanish-Canadian woman (CM), four native Swedish women (AW, MKA, ECL and HK) and one woman from Somalia (SS). AW, MKA, HK and SS are all midwives with clinical experience in contraceptive services. The entire team was trained in qualitative methodology and had both clinical and research experience with SRHR and sensitive topics, including migration. The team was aware of how their lived experiences could affect the research process, data collection, and analysis^44^ and accepted responsibility for interpreting the data, recognising how their realities, preconceptions, and knowledge might influence the findings. During the process, doctoral student AW wrote a reflexive journal, and the team reflected on power relations through iterative discussions about their positionality towards the topic, the participants, and the project.

### Ethical considerations

Ethical permission was granted by the Swedish Ethical Review Authority (Dnr 2020-05710, 2021-01-07, and Dnr 2021-06007-02, 2021-12-05). The study followed the guidelines of the Declaration of Helsinki.^45^ Each participant received both verbal and written information about the study, and all who agreed to participate signed an informed consent form or provided a digitally recorded verbal consent before data collection began. All participants were assured that their information would remain confidential and that they could withdraw from the study at any time without explanation. None of the participants withdrew.

## Results

Two main themes were constructed during the analysis process: (1) Factors shaping reproductive choices: community, household, and partner influences and (2) Women’s perspectives on contraceptive services. Each theme comprises subthemes that elaborate on the theme (Table 3).

**Table 3:** Overview of themes and subthemes

Themes	Subthemes
Factors shaping reproductive choices: community, household, and partner influences	- Community influences on reproductive information sharing and decision-making - Adapting reproductive intentions in response to migration to Sweden
Women’s perspectives on contraceptive services	- Barriers to inclusive and responsive services - Calls for active inclusion in shaping contraceptive services

### Factors shaping reproductive choices: community, household, and partner influences

This theme illustrates how reproductive choices were shaped by multiple, interconnected influences that extended beyond individual preference. Community knowledge and oral tradition played a central role in circulating reproductive information and guiding decision-making. Family and partner dynamics within the household also influenced how decisions were negotiated. Migration to Sweden introduced new social and economic circumstances that prompted participants to reconsider intentions regarding family size and contraceptive use. Religion remained a significant influence, although its interpretation varied according to the context and personal circumstances.

#### Community influences on reproductive information sharing and decision-making

The community was described as a source of reproductive information and advice. Discussions with friends, other women, and family members provided knowledge that could support decision-making, but also reinforced social expectations that, at times, limited women’s autonomy.

Participants described the community in this context as a group of Somali-born women of different ages who regularly met to discuss topics related to their lives, including issues such as reproduction and contraception. Discussions and exchange of experiences were considered the preferred method for generating and transmitting knowledge. As one participant explained (FGD 2), “*You know, we are an oral community that communicates and conveys messages to each other constantly”*.

The FGDs reflected these community practices. When a participant shared a difficult or traumatic memory, it often elicited strong reactions from others, as if they had experienced it together. Differences in perspectives on SRHR were typically navigated through collective discussion rather than direct confrontation.

Participants often consulted their community before visiting maternal health clinics, describing this as helpful in decision-making:

“*We consult always with friends and other women and learn from them, the person consult with friends on the different type of contraceptives and which one is the best … So we get many advice from the friends.”* (FGD 3)

However, information from friends could also deter contraceptive use:

“*I wanted to use contraceptives for child spacing and consulted my husband, and I knew from my friends’ discussions that they encountered problems using it, so I decided not to use a contraceptive [method].”* (FGD 4)

Some women described making decisions together with their partners. As one participant summarised it:

*“Me and my husband went to the midwife and took from her the information letter. We read that information and we liked the condom and begun to use it.”* (FGD 4)

Relatives, particularly mothers, were seen as sometimes reinforcing misinformation shaped by community norms and patriarchal expectations that women should prioritise childbearing over their own reproductive choices. As one participant explained (FGD 8), “*When you consult with your mother, she pushes you to have children, so it is better not to share with her”.* Husbands were also sometimes described as exerting pressure. As one woman explained:

*“I believe that it is not the business of the husband because he always wants me to give birth to children, that is the reason he married me. So, I think that the husband has no role in this issue.”* (FGD7)

In response, some described deliberately excluding family and partners from decision-making as a strategy to safeguard their autonomy, while others emphasised asserting independent control over their reproductive choices. As one participant expressed, “*I told my husband … that we will use condom to prevent pregnancy”* (FGD8).

Another person reinforced this sentiment:

*“I am a little bit authoritarian, so I just depend on my own views. If I ask my husband, I think maybe he would like more children, so I do not consult with him … The decision comes from me when I want a child, and he accepts it … so the decision is always mine.”* (FGD 3)

Shared stories played a significant role in affirming women’s reproductive autonomy and in exchanging strategies to resist unwanted influence from partners and families. One participant observed:

*“ … her husband always pressures her to get pregnant. I believe that is not up to the man, it is up to her … because it is related to her health.”* (FGD 7)

Partner involvement in reproductive decision-making was far from uniform. For some, partners and family were excluded due to pressure and coercion, while for others, they were seen as equal participants.

Participants emphasised that decisions about childbearing and contraception should be theirs, free from coercion. Yet autonomy was not exercised in isolation; it was negotiated within social and relational contexts, reflecting the collective nature of reproductive choices in their communities:

*“Regarding contraceptives, you’d take what’s convenient for you and your life situation at that time. The decision is mine, but first I talk to my husband because it is we who are going to take care of the child and it’s our responsibility. Yes, I talk to my friends too, because when we meet, we ask each other questions and exchange ideas.”* (FGD 1)

#### Adapting reproductive intentions in response to migration to Sweden

Migration to Sweden reshaped participants’ reproductive perspectives and contraceptive choices. Everyday circumstances, access to services, and changes in family life influenced their decisions. Religion continued to guide family building, but was often reinterpreted in the new context. Participants described reconsidering the number of children they viewed as ideal once in Sweden, noting that their reproductive needs and contraceptive use sometimes changed with shifting life circumstances. Sweden was regarded as more demanding for family life, as expressed by one woman:

*“The environment [in Somalia] helps you, as you live with your relatives and country. The sun helps you, as that does not require you to buy heavy clothes for the children … But when we came here, we experienced a different country, culture and a different life that is harder than the one we fled from.”* (FGD 3)

Women highlighted challenges such as the cold climate, which required costly clothing and equipment for children, and the absence of extended family support. Material and social constraints heightened the importance of reproductive planning and contraceptive use. Participants spoke of greater awareness of the need to space children and described contraception as essential for protecting their own and their families’ health and well-being. Over time, women reported increased familiarity with both hormonal and non-hormonal methods and acknowledged that contraceptive experiences varied widely. One participant explained how her views shifted:

*“I have seen that my children are increasing in number. … Now, I realise that this is my problem, so I decided to stop being suspicious about contraceptives - if they are bad, then why are Swedish women using them. You do not see many people dying because of them, so I decided to take it and I am satisfied.”* (FGD 3)

Migration history shaped reproductive decision-making in important ways. Newly arrived women were described as facing greater challenges, including limited knowledge, language barriers, and insufficient information about reproductive rights. In contrast, women who had lived in Sweden longer reported greater awareness of reproductive health and contraceptive options, which supported informed choices:

*“So, women are really vulnerable, and they need more help particularly when the person is a newly arrived immigrant. But people who lived long in Sweden, like me, know a lot.”* (FGD 2)

Religion remained significant, though its interpretation varied. Some participants described family size as determined by God and beyond individual control, while others reinterpreted religious principles in the Swedish context, allowing for more deliberate planning:

*“You have to have a plan … before you get the child; it is not time to say God destined [children] to come here … The partners must discuss this before marriage … they have to learn the language. It is also important to have a good house or apartment for residence when they want to have children.”* (FGD 5)

Contrasting views illustrated intragroup variation in how religion influenced reproductive choices and autonomy. Overall, reproductive intentions were shaped through the complex interplay of material realities, migration history, community influences, and religious beliefs. Reproductive decisions were relational, negotiated, and evolving over time in response to life in Sweden.

### Women’s perspectives on contraceptive services

This theme illustrates participants’ willingness to receive contraceptive services and their awareness of their importance. However, participants also described encounters within contraceptive services marked by limited or simplified information, judgmental comments, and stereotypical assumptions, which generated feelings of discrimination. These experiences were rarely isolated; they were reinforced through shared stories within the community, shaping a collective view of contraceptive services as unresponsive. Participants also articulated concrete ways forward, emphasising timing and consent in counselling, access to appointments and follow-up, comprehensive information, community-engaged approaches (e.g. group counselling in Somali), and considered approaches to partner involvement.

#### Barriers to inclusive and responsive services

Participants expressed awareness of the importance of child spacing to support health and well-being. They discussed knowledge of hormonal and non-hormonal methods and highlighted pregnancy as a key moment to obtain information from midwives. Women also described knowing how to navigate contraceptive services. As one participant explained (FGD 2), *“I think that it is good that the midwife speaks with the pregnant mother about child spacing”*. However, participants described a wide range of barriers within contraceptive services. Language was one challenge, but women emphasised that limited or simplified information, judgmental comments, and stereotypical assumptions also undermined the quality of services. Several participants questioned whether the restricted information they received reflected discriminatory attitudes:

*“So, they don’t give good information, I don’t know, is it discrimination against the Somalis? I don’t know, but they are not giving you sufficient information. It also depends on the midwife who meets with you, but it does not depend only on language proficiency.”* (FGD 8)

Such encounters reinforced women’s sense of being treated differently:

*“Yes, they have to stop that because that can make us hate the country because they speak with us as ignorant people who understand nothing. If you speak to a stone does it understand you? They assume you are a person who does not understand anything at all. We have the capacity to manage and raise our children.”* (FGD5)

Comments that assumed pregnancy or preferences for a large family size were particularly damaging. One participant recounted:

“*‘You will come back next year’ [the midwife said]. How do you know that I will be pregnant? How can you say to me that I will be there next year. I will come if I want or will not come if I do not want it – it is my business, not yours. That is something which can create a lack of trust.”* (FGD 8)

Across FGDs, participants described similar statements as insulting and as reinforcing expectations around fertility. Another participant (FGD 2) continued, “*Many women protested against that, it is not only she who encountered it, but many women got angry because of that statement”.* These remarks affected reproductive autonomy by reinforcing assumptions about pregnancy expectations. Participants described these experiences as part of a wider pattern shaping community-level perceptions of the health system.

Seeking contraception was often met with scepticism, based on the assumption of an inevitable desire for more children:

*“They think that you are Somali and want to remove it (the IUD) after short time because you want a child. I mean how she treated and talked with me and how she gave me the information was not good.”* (FGD 8)

The repetition of these experiences across different encounters suggested that such treatment was not limited to individual midwives but may reflect broader systemic patterns within contraceptive services. Institutional norms, rather than individual biases, appeared to shape these encounters. Similarly, questioning about family size positioned them as being treated differently from other clients, framing their reproductive choices through a lens of stereotypes rather than individualised healthcare needs:

*“What she says is correct, I am asked the same question whenever I go to the health centre or meet a midwife. How many children do you have? Where are your children? … Every time I am asked the number of children I have while I am waiting the first child, but still they are asking me.”* (FGD 2)

The shared experiences generated collective stories that discouraged women from seeking contraceptive services, with delayed or missed care described as the most far-reaching consequence. As one participant described:

*“I fear to go there. … It is not good that people fear the health centre, but this is a reality. And you know, it is the place where they would seek help, so if the person fears it, the health problems can increase.”* (FGD 4)

Participants also described how stereotypical assumptions extended to Somali-born men and family dynamics. As one participant reflected:

*“It is good that father accompanies the mother but there are many circumstances that cause the mother to go alone … , but they (midwives) think that mother is alone because she is oppressed. We are a connected family, the mother is not oppressed, and the father cannot do anything without the acceptance of the wife and vice versa.”* (FGD 4)

Women emphasised the importance of their community as a source of collective knowledge and support. Through sharing experiences and strategies, they resisted pressures from both family members and healthcare services, thereby strengthening one another’s capacity to make reproductive decisions on their own terms. This was emphasised by one participant, “*I am telling the Somali woman not to be afraid because the decision to have a baby is yours and the pregnancy is yours (not the midwives)*” (FGD2).

Participants also described how public remarks made by politicians, suggesting that Somali-born women have too many children and rely on state-funded child support, negatively influenced their perceptions of the government’s intentions for offering contraceptive services. As one participant explained:

*“I met a Somali woman who is seventh month of pregnant that did not see her midwife because she was afraid to go there … woman fear meeting with the midwives because of their unnecessary questions or wrong perceptions.”* (FGD 4)

#### Active inclusion in improving contraceptive services

Alongside barriers, women actively shared ideas for how contraceptive services could be improved. They emphasised timing and consent in counselling, access to appointments and follow-up care, and the right to discontinue methods. Women also suggested ways forward, including group counselling in Somali, closer collaboration between services and the community, and discussions on partner involvement. These perspectives highlight women’s agency in shaping services to better meet both individual and collective needs. Community-engaged education and group counselling in Somali were suggested as ways to strengthen understanding and readiness, rather than creating pressure during encounters with contraceptive services. As one participant described:

*“If they want us to make child spacing they have to organise lectures and educate the people and make them understand and become ready for child spacing instead of pressuring a patient who went to the hospital for help to make child spacing. That can help us to plan making child spacing so that we adopt with the country, but they have to stop the mistreatment against us.”* (FGD 5)

Timing, consent and flexibility were described as prerequisites for improved contraceptive services. Women emphasised that services must respect their right to decide when and how to receive information. When timing was imposed, counselling was experienced as pressure rather than support. Participants felt that contraceptive conversations during antenatal visits sometimes prioritised avoiding future pregnancies over care for the ongoing one.

The participants stated that they owned their bodies and should have the final say in reproductive decision-making *“You are a mother, mature person, and you have to decide it, the midwife has not right to impose her decision on you”.* (FGD3) Another explained, *“they (midwives) are deciding what you have to do, I say to them I am free if I have a child or not, it is my decision”* (FGD3). One woman added:

*“I believe that I was forced to take these pills. I became sick when I used the pills and then contacted with the midwife, but she said continue it.”* (FGD 2)

Being unable to decide on the timing of counselling created negative feelings and was described as coercive. Participants stressed that consent related not only to the content of counselling but also to when it was provided. As a way forward, they recommended dialogue and clear explanations as prerequisites for voluntary counselling:

*“I have the pressure of this pregnancy. I am worried about my delivery and whether I will deliver on time or not. And in that situation, they are speaking with you about a non-existent and future child. We will not be upset if they respect our freedom; ask us if we want information and whether we want to make child spacing or not.”* (FGD 3)

Improving contraceptive services included listening to women’s experiences and ideas. Participants emphasised the need for comprehensive information and tailored counselling, including both hormonal and non-hormonal methods and transparency regarding side effects. They also emphasised antenatal visits as an opportunity for such discussions and advocated collaboration with the community in the design of contraceptive services.

Partner involvement was also discussed. Midwives were described as encouraging shared reproductive responsibility between partners, and couples’ visits were seen as a way to ensure both were informed about options, side effects, and mutual responsibility. However, views differed: some wanted their partners included, while others preferred to exclude them to safeguard their own autonomy. Despite differing views, some participants framed involvement as an underused strategy to improve services and shared understanding:

*“I would say that it should be a requirement that the husband accompany his wife to the appointment scheduled for the woman to take a contraceptive, such as an IUD, so that he knows the information about the benefits and side effects.”* (FGD 8)

Participants also highlighted barriers to accessing contraceptive services. Missed opportunities for IUD insertion were linked to difficulties in collecting the device, limited appointment availability, or the need to schedule a separate visit for insertion. Limited access to follow-up visits when experiencing side effects created fear:

*“The other formulas are worse than the pills because pills are something you can manage, but they are prolonging the appointment when you are using the other method (IUD) … and the person wants the IUD removal, but the pills are easy to use. I have seen many girls who are complaining from that and did not get appointments when they had bleeding and wanted to remove it.”* (FGD 2)

These barriers, rooted in both encounters within contraceptive services and wider health system arrangements, influenced method choice, as women sometimes avoided methods they felt they could not control. Together with discriminatory encounters, these barriers reinforced negative perceptions of the health system. To address these challenges, participants recommended fostering dialogue, challenging generalised assumptions among healthcare providers, and enhancing communication between the health system and the Somali community.

## Discussion

Our main findings reveal a willingness to receive contraceptive services and awareness of the importance of child spacing. However, barriers to reproductive justice in contraceptive services were obvious, highlighting structures in the health system that may influence the health and well-being of Somali-born women in Sweden. The most prominent finding was the experiences of prejudice and discrimination, which affected contraceptive decision-making and access to services. Participants described situations where they felt racially profiled based on assumptions about high birth rates and reluctance to use contraception. This aligns with studies in other high-income countries^46^ showing that discrimination during healthcare encounters^47,48^ undermines reproductive autonomy and justice for immigrant women.^49,50^ Participants expressed a strong desire for control and involvement in contraceptive decision-making, but provider prejudice and lack of respect for their values constrained this autonomy. These findings echo a scoping review on discrimination in maternal healthcare^50^ and underscore the need for culturally safe services that actively work to recognise and reduce bias.

Shared experiences were central. However, women’s narratives also highlighted important differences shaped by migration histories, gender relations, religion, and socioeconomic positions. This reflects the intersectional grounding of reproductive justice, which situates reproductive decision-making within multiple, overlapping systems of power.^24,28^ These variations underscore that Somali-born women are not a homogeneous group, and that reproductive issues are negotiated at the intersection of intimate and relational contexts, as well as structural constraints (e.g. language, health literacy, service delivery, provider approaches) that limit reproductive autonomy, as also noted in similar settings.^5^

Partner involvement was viewed as potentially supportive, particularly for promoting shared reproductive responsibility and increasing male engagement in SRH, consistent with research on male involvement.^51^ However, participants held diverse views, also consistent with previous research.^52^ While Somali-born women are sometimes portrayed as dependent on their partners,^53^ studies indicate that communication and shared responsibility are common, and women often make final decisions independently,^46,52,54^ a pattern confirmed in this study. Partner involvement can support reproductive justice when it is consensual, confidential, and respects women’s autonomy.^54^

Our findings also align with the concept of contraceptive justice.^55^ This framework emphasises person-led, non-coercive and bias-aware services, grounded in informed choice. In our study, women highlighted the importance of explicit consent and appropriate timing in contraceptive services, showing how services that were not aligned with their needs risked undermining autonomy. Experiences of stereotyping and assumptions about Somali women’s fertility illustrate how implicit bias obstructs person-centred care and limits reproductive choices and autonomy. At the same time, women described their community as a crucial space for sharing knowledge and strategies, which helped them resist pressures from both families and services. This collective agency expanded the ability to make reproductive decisions on their own terms and points to the need for services that recognise community-based resources as part of advancing reproductive justice in practice. Recognising how women negotiated gendered power relations within Somali families can further inform the design of culturally safe and equitable maternal health services, fostering trust and improving the quality of care.

Contrary to previous studies suggesting immigrant women lack reproductive health knowledge,^56,57^ our participants demonstrated awareness of their needs. Findings indicate that health system barriers, not only individual deficits and healthcare service encounters, are central. As Sweden becomes increasingly diverse, the health system must prioritise cultural safety, client experience, and flexible service arrangements.^54,57–59^

Prior research highlights Somali women’s preference for group counselling and community-based approaches^58,60,61^ that incorporate community knowledge as a strategy to advance reproductive justice.^24,28^ In Sweden, contraceptive services are streamlined to prioritise shorter visits^9,62^ and to increase digital counselling and remote prescribing.^59,63^ While such efficiency may enhance accessibility, our findings highlight that it can also risk marginalising women’s voices, underscoring the need for including person-centred and justice-oriented approaches. From a reproductive justice perspective, participants’ experiences illustrate how structural inequities in service organisation limit access to group counselling and flexible appointments.

The timing of contraceptive services was central to participants’ experiences. Informed choice – including support for consent to counselling and for choosing or discontinuing methods – emerged as a core value. While this reflects reproductive autonomy and reproductive rights, participants’ accounts also show that these rights depend on structural conditions, such as the organisation and delivery of services. A recent Swedish study found that immigrant women report higher levels of negative mental health side effects from contraceptives compared to Swedish-born women.^64^ This finding underscores that the lack of comprehensive discussions regarding side effects and follow-up is not a minor clinical issue, but a structural concern that shapes women’s willingness and ability to use certain methods. In our study, participants’ mistrust was linked not only to the timing of counselling but also to uncertainty about whether they would receive support if they experienced side effects.

Participants also emphasised the importance of belonging to what one participant described as an “oral community” for sharing reproductive knowledge, which further affected their decision-making and autonomy. Honouring lived experience and knowledge traditions, by taking women’s own accounts and community knowledge seriously in contraceptive counselling, is essential to designing person-centred care that supports reproductive justice.^24,50,55,65,66^ While women described mistrust arising from individual encounters within contraceptive services, our analysis shows that these recurring and collectively recognised experiences reflected structural patterns in the health system, where system-level arrangements undermined reproductive autonomy and equity. This mistrust must be understood as grounded in intersecting systems of oppression, including racism and xenophobia in Swedish healthcare services and the overall health system,^50,67–70^ as well as patriarchal assumptions about authority in reproductive decision-making,^29^ and efficiency-driven service arrangements such as limited appointment availability, delayed or separate visits for IUD insertion or removal, and insufficient follow-up.^71,72^ Addressing this mistrust requires accountability at multiple levels, from clinical encounters to health system governance, to ensure that reproductive justice principles are translated into everyday practice.^24^ Several women also linked their experiences to political discourses portraying immigrant women as having “too many children.” As Mulinari et al. (2023)^73^ show, Swedish reproductive health and population policies have been shaped by reproductive racism, where some groups were targeted for fertility limitation while others were encouraged to have more children.^73^ This resonates with public debates problematising higher fertility rates among women in socioeconomically marginalised areas,^74,75^ while at the same time, the Swedish government has launched an inquiry into how fertility can be increased nationally.^76^ Political debates about limiting childbirth in specific areas have further eroded trust in Swedish contraceptive services. Such seemingly contradictory discourses illustrate how nationalism, migration politics, and demographic governance intersect to produce inequities in Swedish reproductive healthcare services. Seen in this light, participants’ sense of being under surveillance was not only personal but reflected a wider political framing of reproduction that risks undermining trust, reproductive autonomy and justice.^24,28^

### Solutions and way forward

This study’s participants expressed a desire to contribute to improving contraceptive services both in clinical encounters and at the system level. As Cadena et al. (2022)^55^ argue in their framework of contraceptive justice, explicit consent to counselling is essential to counter power imbalances and ensure person-led, non-coercive services. Participants echoed this by emphasising that each counselling session should begin with a mutual agreement on whether and how contraception should be discussed. In this way, consent acted not only as an interpersonal practice but also as a structural safeguard of self-determination in SRHR.^34,50,55^

Participants proposed several concrete solutions to improve service. They emphasised the value of group counselling in their native language and advocated for opportunistic counselling during visits rather than deferring care due to time constraints. Difficulties in booking appointments for IUD removals were highlighted as barriers undermining trust in these methods. These insights indicate the need for greater flexibility and responsiveness across the health system.

The emphasis on timing and consent also aligns with established frameworks for sexual health counselling, often used in the Swedish healthcare services. The PLISSIT framework (Permission, Limited Information, Specific Suggestions and Intensive Therapy)^77^ and its extended version, EX-PLISSIT,^78^ highlight the importance of permission at every stage and provider self-reflection to redress power dynamics in services. In line with contraceptive justice,^55^ systemic changes in governance, policy, education, and accountability^66,79^ are needed to achieve more equitable services.

Advancing reproductive justice further requires embedding clients’ values and needs into contraceptive services,^24,55^ promoting ownership of reproductive choices and reducing the influence of provider preconceptions, an ongoing challenge in the Swedish health system.^67,80^ While our findings highlight systemic and interpersonal barriers, it is also important to recognise how participants exercised agency and resistance within constrained contexts by asserting preferences, by sharing community knowledge, and by challenging provider authority. These actions extend beyond individual choice; they represent strategic negotiations of power within the health system. At the same time, we acknowledge that these expressions of agency may not capture the full diversity of experiences across the broader Somali-born population. Future research should further investigate how overlapping factors, such as age, time since migration, and socioeconomic position, influence access to services and reproductive autonomy.

### Strengths and limitations

A strength of this study was that 60 women participated in FGDs and actively shared their perspectives. Having a Somali-speaking moderator was crucial for creating a safe space where participants felt comfortable engaging, agreeing, and disagreeing, and it likely contributed to their willingness to participate in member reflection. Conducting the FGDs in Somali also enabled richer dialogue, although translation into English posed semantic challenges. This was addressed through reflection within the research team and by checking the meaning of key phrases, as one researcher is bilingual. To strengthen transparency, we exemplified the analytic process through an audit trail and presented quotes to illustrate the relationship between transcripts and findings (Figure 1).

The participants had considerable experience with Swedish healthcare, including antenatal care and follow-ups, and many were involved in local outreach activities. While this provided valuable insights, it also meant that recently arrived women or those less active in the community were not represented. Somali-born women are not a homogeneous group, as reproductive decisions are influenced by socio-economic background, education, age, and migration history. Another limitation is that the study does not include midwives’ perspectives, although these are addressed within the wider Improve-it project.^10,59^

## Conclusion

The study findings suggest opportunities to advance reproductive justice in contraceptive services in Sweden. Participants expressed a readiness to engage in discussions about their reproductive health, emphasising that incorporating explicit consent into contraceptive counselling would improve the acceptability of services. Addressing prejudice and undue influence in both the health system and clinical encounters is critical. There is an urgent need to raise awareness among healthcare providers and policy-makers that Somali-born women are ready to participate actively in decisions about their reproductive health. Incorporating women’s preferences and needs is essential to establishing person-centred contraceptive services, aligning with Sweden’s health system priorities. Services must adapt to and reflect clients’ lived experiences to avoid being shaped by preconceived notions and intersecting power dynamics.

## Supplementary Material

Supplemental Material. COREQ Checklist

Supplemental File 1. Topicguide FGD.

## Data Availability

A protocol has been published prior to this project [10]. Data included in this study are available upon request.

## Provenance statement

This article was not commissioned and went through external peer review.
