# Supplementary material for: Exploring Somali-born women’s experiences with contraceptive services in Sweden through a reproductive justice lens
Source: Sex Reprod Health Matters. 2026 May 8;33(1):2667110. doi: 10.1080/26410397.2026.2667110 (PMC13276817; doi:10.1080/26410397.2026.2667110)
Supplement: Supplemental File 1. Topicguide FGD. [file ZRHM_A_2667110_SM1958.docx]

Themes:

-pregnancy planning linked to

-contraception,

-knowledge/use,

-partners’ involvement,

-access,

-desired information and approach regarding counselling (where, when, how, by whom) linked to pregnancy.

Moderator:

Probes:

Can you tell me more?

Can you develop?

Can you give examples?

What do you think about this?

Do you recognise this, anyone with different experiences?

Have you heard about other experiences?

ADD PROBE GENERAL: Other thoughts?
